# #OMFSurgery: analyzing the use of social media applications in oral and maxillofacial surgery resident training

**DOI:** 10.1186/s12903-023-02872-9

**Published:** 2023-04-12

**Authors:** Lukas B. Seifert, Philipp Becker, Andreas Pabst, Anna K. Sander, Josephine Schneider, Lara Schorn, Alexander Zeller, Jürgen Hoffmann, Daniel G. E. Thiem

**Affiliations:** 1 Department of Oral, Cranio-Maxillofacial and Facial Plastic Surgery, Goethe University, University Hospital Frankfurt, Theodor-Stern-Kai 7, 60528 Frankfurt Am Main, Germany; 2 Department of Oral and Maxillofacial Surgery, University Medical Center Mainz, Augustusplatz 2, 55131 Mainz, Germany; 3 Department of Oral and Maxillofacial Surgery, Federal Armed Forces Hospital, Rübenacherstr. 170, 56072 Koblenz, Germany; 4 Department of Oral and Maxillofacial Surgery, University Hospital Leipzig, Liebigstr. 12, 04103 Leipzig, Germany; 5 Department of Oral and Maxillofacial Surgery, Hannover Medical School, Carl-Neuberg-Straße 1, 30625 Hannover, Germany; 6 Department of Oral and Maxillofacial Surgery, University Hospital Düsseldorf, Moorenstr. 5, 40225 Düsseldorf, Germany; 7 Department of Oral and Maxillofacial Surgery, University Hospital Heidelberg, Im Neuenheimer Feld 400, 69120 Heidelberg, Germany

**Keywords:** Oral and maxillofacial surgery, Social media, Training, Education

## Abstract

**Background:**

Despite its increasing popularity, to our knowledge the use of social media applications (SM) for residents’ training in Oral and Maxillofacial Surgery (OMFS) has not been investigated yet. The aim of this study was to evaluate the use of SM applications by OMFS residents for post-graduate training in Germany.

**Methods:**

For explorative assessment, an online questionnaire containing 27 questions about the current use of SM for resident training was sent to OMFS residents in Germany.

**Results:**

Sixty-four colleagues participated to the study. Thirty-four participants (54%) responded to regularly use those platforms mainly for OMFS-related content. YouTube (65%, *n* = 37), Instagram (48%, *n* = 27), ResearchGate (25%, *n* = 14) and WhatsApp (16%, *n* = 9) were the most popular platforms. (Surgical) videos (97%, *n* = 59), pictures and graphics (82%, *n* = 50) were the mainly accessed contents. Forty-four participants (69%) stated that SM substantially contributed to their OMFS training. Dentoalveolar surgery and implantology (66%, *n* = 35) and aesthetic facial surgery (55%, *n* = 29) content contributed most to OMFS resident training. Fifty-one participants (80%) recommended an official SM account of the DGMKG.

**Conclusions:**

SM is frequently used by OMFS residents for the consumption of training-related content. There is an imbalance toward dentoalveolar and facial aesthetic surgery regarding the presented content. Academic institutions and societies should complement their educational activities to not miss this emerging educational innovation. Official SM content by academic institutions and societies could contribute to the existing educational activities.

## Background

The use of social media applications (SM) has become an integral part of everyday life for a majority of people worldwide. In this context, the term “going viral” describes the enormous expansion of information by SM. In the US, around seven out of ten people are using SM to connect with their peers, share information or for entertainment purposes [1]. Among the most frequently used platforms such as Facebook, Twitter or Instagram especially the latter has grown tremendously since its introduction in 2010. Today, Instagram has over 1 billion active users and boasts an engagement level which is ten-times higher than Facebook and thirty-times higher than Twitter [2]. As a logical consequence, many industries, including the healthcare industry, are increasingly using SM platforms such as Instagram for marketing, advertising and health promotion [3, 4]. Despite initial concerns by the medical field about SM and possible legal issues and implications for professionalism [5], many medical professionals, predominantly from the field of plastic surgery and dermatology, increasingly use their SM presence for marketing purposes and health promotion [6]. Some physicians even attained massive public attention among people outside the medical field and made a career by early on sharing surgical videos on SM platforms, like the famous dermatologist Dr. Sandra Lee (Dr. Pimplepopper) with over 4 million followers [7]. In the field of oral and maxillofacial surgery, numerous SM accounts have emerged in the recent years enjoying increasing popularity, e.g., the account of Dr. Jason Auerbach (Bloodytoothguy) who regularly posts educational videos of his surgical procedures and who now has more than 175,000 followers [8]. Against the background of the ongoing SM success mainly experienced by private practitioners more and more academic institutions and societies are starting their own SM accounts for self-promotion, talent-recruitment and presentation of their residency programs [9]. However, a recent study by Yang et al. found that the number of OMFS residency programs represented on SM is growing but is still significantly lower than that in other surgical fields, such as plastic- and reconstructive surgery, which might lead to missed opportunities for promotion and delivering information to trainees [10].

Although there are studies that report SM to be a valuable tool for undergraduate dental education [11], there is a lack of information about the educational value of SM for post-graduate OMFS training. The aim of this study was to explore the educational value of SM for post-graduate training within a nationwide survey among OMFS residents in Germany.

## Methods

### Questionnaire design

For an explorative assessment of SM use among residents for post-graduate training in OMFS in Germany, an online questionnaire including 27 questions was designed and prepared in German language (Table 1) by the authors of this study (Young Forum^1^ of the German Association of Oral and Maxillofacial Surgery). For this purpose, based on a literature review the study group identified key questions regarding the study's topic. Possible questions were selected through group discussions and evaluated to determine their suitability for further use. The criteria for selection were comprehensibility, accuracy, and specificity regarding the topic of interest. Consensus on issue was achieved through group discussion. Free-text and multiple-choice questions were used. The effects were estimated using a five-point rating scale. After internal validation, the questionnaires were presented to clinicians familiar with educational studies for external validation. Issues found were resolved by adapting the questionnaires to address them. To avoid drop-out due to fatigue, the questionnaire was designed to be completed in less than five minutes. The basic demographics were initially asked.

**Table 1:** Topics in the questionnaire

Topic	Question	Optional questions	Answer
Demographics	Gender	Male Female Diverse	SC
	Year of training	1 2 3 4 5 Board certified CMF-surgeon	SC
	Degree	Medical Dentistry Dual degree	SC
	Which subfield of maxillofacial surgery is your focus of interest?		MC
		Oncologic surgery	
		Traumatology	
		Dentoalveolar surgery	
		Orthognathic surgery	
		Facial esthetic surgery	
		Reconstructive surgery	
		Surgery of malformations	
	Which devices do you mainly use for "social media" applications?	Smartphone Notebook Desktop PC Tablet	MC
	Where do you primarily use social media?	At work At work On the way	MC
	For what purposes do you mainly use social media?	Entertainment Inspiration Education Peer exchange	MC
	What is the estimated amount of time you spend on social media platforms weekly?	0 – 6 h 7 – 15 h 16 – 24 h 25 – 40 h > 40 h	SC
	Of that time, how much do you use social media for professional purposes?	None < 25% < 50% < 75% > 75%	SC
	Which social media platforms do you mainly use?	Facebook Instagram YouTube WhatsApp Twitter LinkedIn ResearchGate Pinterest TikTok Siilo	MC
	Which "social media" platforms do you primarily use to access content relevant to continuing education in the field?	Facebook Instagram YouTube WhatsApp Twitter LinkedIn ResearchGate Pinterest TikTok Siilo None	MC
	What type of media do you use on "social media" platforms to access education-related content?	Images (Surgical)-Videos Chat forums Scientific publications Guidelines Simulation training Lecture references None	MC
	Do you think that content relevant to professional education on "social media" platforms has contributed to your education so far?	Yes I don’t know No	SC
	If yes, how much has professional-related content on "social media" platforms already contributed to your education?	None < 25% < 50% < 75% > 75%	SC
	If yes, in what area has using social media contributed most to your professional education?	Oncologic surgery Traumatology Dentoalveolar surgery Facial esthetic surgery Reconstructive surgery Surgery of malformations Others	MC
	What are reasons for you to use social media (SM) as a professional training platform compared to traditional education platforms (books, congresses, training courses, etc.)?	SM is easier to use Posting of answers and questions Access via multiple devices Free of charge Intercollegiate communication Global access Fast search Only additional usage Interactive Access to video material Fast and easy access	
	Are there any concerns on your part that would hold you back from using social media as a source of professional education?	Patient privacy concerns Concerns about the content validity No concerns Fake news Little knowledge transfer	
	Would you like to see content relevant to professional education through an official "social media" channel of the DGMKG?	Yes I don’t know No	
	If you would like to have a DGMKG social media channel, on which platform?	Facebook Instagram YouTube WhatsApp Twitter LinkedIn ResearchGate Pinterest TikTok	
	If yes, what kind of content do you would like to see?	Images Surgical videos Chat forums Scientific publications Official guidelines Simulations training Events	
	Have you already had internal training on "How do I use social media?" in your professional training?	Yes I don’t know No	

*FT* Free text, *MC* Multiple Choice, *NRD* Numeric rating by dropdown menu, *NRS* Numeric rating scale

### Survey administration

The survey was conducted using the Google Forms® tool for online questionnaires in German language in a one-stage assessment. Contact data of the study population were retrieved from the nationwide registry of the German Association of Oral and Maxillofacial Surgery (Deutsche Gesellschaft für Mund-, Kiefer- und Gesichtschirurgie, DGMKG). Residents listed in the DGMKG registry were identified to become study participants and approached via email. After two weeks, a reminder was sent to the non-responder group. Data were anonymized and output using Google Forms® in a Microsoft Excel ® sheet (Microsoft Corporation, Redmond, WA). Hereafter, the data were stored separately. After data export, the answers could not be traced back to the participants.

The study was conducted according to the principles of the Declaration of Helsinki. Informed consent to participate was retrieved from all participants prior to study conduction.

### Statistical analysis

Statistical analysis was performed descriptively using the mean and standard deviation of the mean or median (when appropriate) using Microsoft Excel ®, Microsoft Corporation, Redmond, WA. Furthermore, the sample size needed to achieve representative results from the survey was calculated a priori with the following settings: assumed population size of all OMFS residents throughout Germany (240), confidence level (95%), margin of error (10%) which resulted in a sample size of *n* = 69.

## Results

A total of 181 members of the German Association of Oral and Maxillofacial Surgery were contacted by email via the Young Forum mailing list with a request to participate in the survey. A total of 64 participants (*n* = 40 male, 64%; *n* = 24 female, 36%) responded and fulfilled the questionnaire completely, which resulted in an overall response rate of 35%, which is slightly underpowered since the statistical Power analysis showed a The participants' level of professional training was as follows: 21% (*n* = 13) were in their first, 19% (*n* = 12) in their second, 14% (*n* = 9) in their third, 11% (*n* = 7) in their fourth, 23% (*n* = 15) in their fifth and final year of OMFS training and seven (11%) of the participants were full-board certified OMFS. Among the 64 participants, 13% (n = 8) held a single medical, 11% (*n* = 7) held a single dental and 76% (*n* = 48) held a dual-degree in medicine and dentistry. When asked about their main clinical interest, 46% (*n* = 29) of participants voted for oncological surgery, 49% (*n* = 31) put their focus on facial traumatology, 36% (*n* = 23) expressed increased interest in dentoalveolar surgery and implantology, 57% (*n* = 36) where most interested in facial plastic and esthetic surgery and 46% (*n* = 29) in orthognatic surgery. Only 17% (*n* = 11) of the participants were interested in reconstructive surgery and surgery of malformations.

### Use of and opinions about social media

**Fig. 1:**
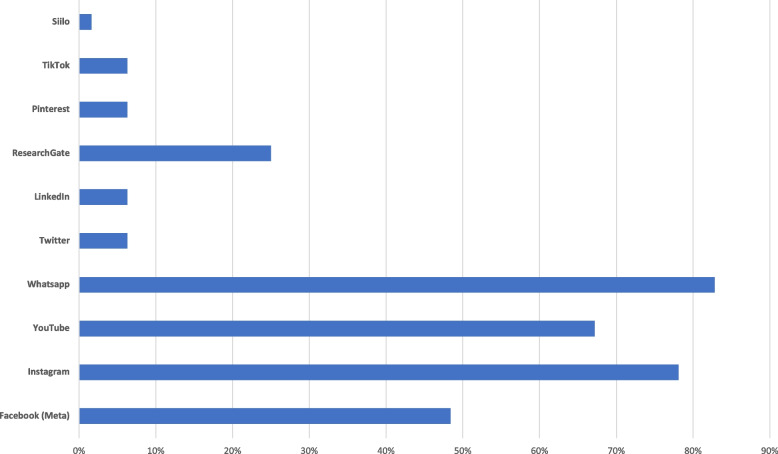
Bar chart showing user popularity among survey participants (multiple answers were possible)

Ninety-eight percent (*n* = 62) of the participants stated that they use their smartphone as their primary device for social media applications. This was followed by notebooks (24%, *n* = 15), tablets (22%, *n* = 14) and desktop computers (9%, *n* = 6). The main place for social media use was cited by 82% (n = 52) as the home environment, 68% (*n* = 43) used social media while traveling, and 17% (*n* = 11) while at work. In terms of primary purpose for using social media, 94% (*n* = 59) of participants selected entertainment as the reason, 65% (*n* = 41) to be inspired, 54% (*n* = 34) for education purposes, and 30% (*n* = 19) for cross-collegiate exchange. Time spent on social media platforms was estimated to be 7 to 15 h by 45% (*n* = 29), 0 to 6 h by 39% (*n* = 25), 16–24 h by 12% (*n* = 8), and between 24 and 40 h per week by 3% (*n* = 2). The proportion used for professional purposes was estimated by 67% (*n* = 43) to be < 25%, 23% (*n* = 15) to be < 50%, and 5% (*n* = 3) each to be < 75% and 0%, respectively (Fig. 1).

**Fig. 2:**
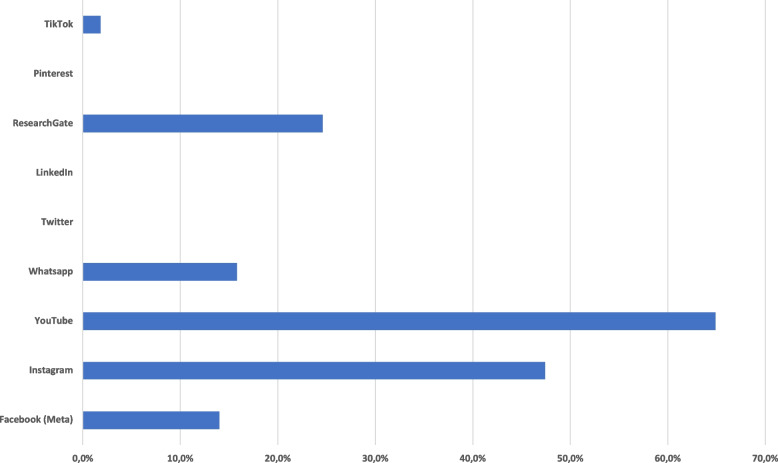
Bar chart showing utilization frequency of different platforms for educational content

To access educational content, 65% used the online video portal YouTube, 47% accessed the social network Instagram, 25% were using the platform ResearchGate, and 14% used Facebook (Fig. 2).

**Fig. 3:**
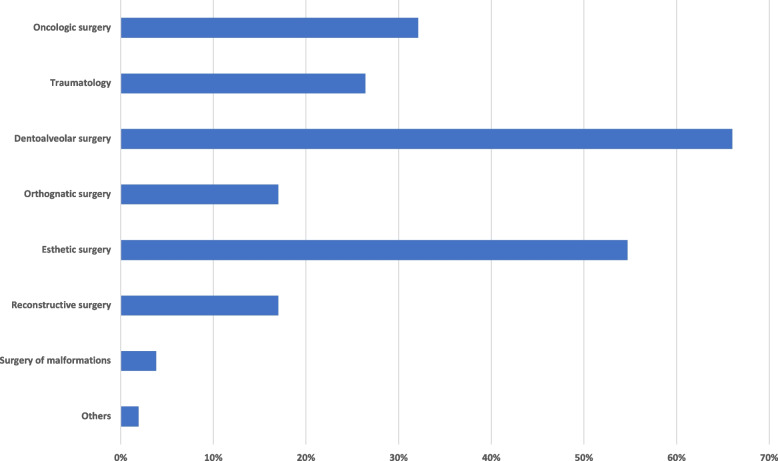
Bar chart shows the subject areas with the most influence on education

In response to the question of what type of media (e.g., videos, images, text) participants primarily accessed on social media platforms, 97% (*n* = 59) selected (surgery)-videos, 82% (*n* = 50) selected images, and 46% each (*n* = 28) accessed scientific publications or guidelines. Sixty percent (*n* = 38) of survey participants agreed with the statement that social media platforms have contributed to their education so far. Thirteen percent (*n* = 8) of the participants were not sure, and 27% (*n* = 17) of the participants disagreed. In the case of a positive assessment, 46% (*n* = 26) of the respondents stated a moderate influence on their education, 23% (*n* = 13) rated the influence as low, 21% (*n* = 12) considered it high, 7% (*n* = 4) saw no influence and only 2% (*n* = 1) a very high influence. As the most represented topics, 66% of the participants named dentoalveolar surgery, 55% facial aesthetic surgery, 32% oncologic surgery, 26% traumatology, and 17% each orthognathic and reconstructive surgery (Fig. 3).

Next, participants were asked to give reasons for using social media instead of conventional media such as textbooks to learn educational content. The main reasons were the simplicity of use (83%), free access (70%), possible use on different devices, and global distribution with international content (55% each). When using medical content on social media, participants had concerns about the accuracy of the provided content (51%, *n* = 31), 43% (*n* = 26) of participants had no concerns, and 30% (*n* = 18) of participants had patient-related privacy concerns. In the following, the participants' desire for a social media channel being offered by the German Association of Oral and Maxillofacial Surgery was surveyed. It was found that 89% (*n* = 55) of the participants would welcome a social media channel. However, 6% (*n* = 4) had no opinion and a further 5% (*n* = 3) rejected the idea of a social media channel. As the preferred platform for a DGMKG social media channel, Instagram ranked first with 77%, followed by YouTube with 59% and Facebook with 20%. With regard to the favored content, surgical videos ranked first with 30% (*n* = 53), followed by clinical images with 23% (*n* = 41), scientific publications with 17% (*n* = 30), official guidelines with 15% (*n* = 26), simulation training with 8% (*n* = 15), and chat forums with 7% (*n* = 12). Regarding the question of whether the survey participants had already received in-house education on the topic of social media use in a professional context, 97% (*n* = 60) stated that they had not received any further education, one participant was unsure, and another participant confirmed participation.

## Discussion

With a response rate of 35%, the study was slightly underpowered, yet comparable to other studies conducted within the collective of the Young Forum of the DGMKG and, thus, was considered as acceptable [12, 13].

Social media has gained increasing importance in the field of medical education in general. Due to increasing digitalization, teaching and organization of education has undergone essential changes [14]. The COVID-19 pandemic accelerated the drive toward digitalization of medical education globally [15]. Social media, in particular, provides unique opportunities for the healthcare industry, serving as a communication medium, marketing tool, and source of data [16]. Because of its heavy use in private settings, social media becomes more and more important in medical education as well. In 2017, an international study showed that 75% of students used social media in private, whilst 20% used it for educational purposes [17]. In Germany, social media is used extensively in medical schools. Seventy-three percent of medical students use social media for educative purposes [18]. Only 54% of OMFS residents use social media for educational content. Social media enhances medical education in terms of real-time communication, interaction with experts and increased creativity [19]. However, social media still plays no formal part of medical education. This is not only because medical education is, in general, taught clinically, very conservatively and traditionally but also because of concerns in data privacy [20]. For OMFS residents, privacy settings seemed relevant to 30% of participants. Protection of data privacy is, especially in Germany, a very sensitive topic, which is hard to combine with privacy settings of most social media platforms. Ethics, privacy and a certain code of behavior are necessary to include social media, chat tools in particular, into medical education [21].

In terms of continuing medical education (CME), industry-sponsored CME and marketing is dominantly available. A certain bias is suspected in these educational materials [22, 23]. Rising The use of social media may offer non-industry-sponsored, evidence-based CME to physicians. In this context McGowan et al. conducted a survey on the daily use of social media by clinicians in terms of lifelong learning and found that 70% of clinicians use social media daily to obtain clinically relevant information. Most participants (57.9%) perceived social media as a good way to get current high-quality information. Moreover, 60% of participants stated that social enabled them to care for patients more effectively [24]. However, a study by Flynn et al. showed that social media only modestly drives physicians to evidence-based CME options. In 2017, out of all tested social media platforms (email, Twitter, Facebook), Facebook offered highest click through rates. Although reaching audience via Facebook appears better than by e-mail, Facebook regulations limit physician organizations to target its members [25]. OMFS residents mainly use YouTube (65%), Instagram (48%), ResearchGate (25%) and WhatsApp (16%) to access OMFS-related content. Facebook, therefore, does not seem too relevant. However, as Instagram and WhatsApp belong to Facebook, regulations apply for these platforms as well. Subsequently, 51% of OMFS residents held concerns regarding the accuracy of the provided content. Considering residency programs, this study shows demand for a social media presence of the German Association of Oral and Maxillofacial Surgery (DGMKG). Eighty-four percent welcomed a social media channel. This matches international results. In the US, for example, only 29.7% of otolaryngological residency programs have social media profiles. This may prevent an opportunity to increase communication with the public via these technologies and to offer evidence-based post-graduate education.

During the last years there has been an increasing number of social media accounts created by surgical departments with the purpose of transmitting educational content to surgical residents. This development might be partly due to the COVID-19 pandemic and the measures of social distancing it brought along [9, 26]. Current studies by Yang et al. evaluated the existence, activity (number of posts, engagement rate, number of accounts followed. etc.) and number of followers of Instagram accounts of all residency programs in oral and maxillofacial surgery in the United States. While, in 2020, fewer than 20% of programs utilized Instagram, an exponential growth was demonstrated during the second half of 2020, with more than 50% of all residency programs managing an Instagram account in January 2021. This was mainly attributed to the urgent need for rapid virtual communication in the COVID-19 pandemic. However, compared to other related surgical disciplines, e.g., plastic surgery, experience with and use of social media seems to be significantly lower in oral and maxillofacial surgery [10, 27].

Because the potential benefits are evident and the development has now been accelerated, we should anticipate a constant further increase of social media use in Oral and Maxillofacial Surgery residency worldwide. It is essential to closely monitor content and utilization strategies to avoid medicolegal problems and create discipline-specific concepts [28, 29].

The currently lower use of social media in oral and maxillofacial training compared to other surgical disciplines might be partly due to the sensitive area of surgery that makes anonymization harder. Another reason might be the highly specialized field that is represented in a smaller number of faculties and clinics. Still, it seems manageable to overcome these obstacles, for example, by not showing identifiable patient information/obtaining patients' consent or by collaborating with other centers to produce adequate content. For various reasons, it should also be rewarding: 1) social media is a feasible way to improve surgeons' education [30]; 2) clinicians and researchers already effectively use social media in different ways as a data source and to stay informed concerning the latest research results; In this context, Sedrak et al. could demonstrate how clinicians and researchers can effectively implement social media channels as a source of clinically relevant information and engage with their community regarding the latest research [31]; 3) during the COVID-19 pandemic experiences in teaching demonstrated that content regarding oral and maxillofacial surgery can be conveyed virtually in a successful manner [32, 33]; 4) experiences in other disciplines have shown that legal, professional and ethical violations are low [29].

It is, therefore, evident, that the use of social media in oral and maxillofacial training will provide a useful addition to surgeons' education once the relevant questions are settled.

Despite the many benefits of social media in resident training, there are some disadvantages, risks, and concerns that may affect the practitioner, the patient, or both. A distinction is made as to whether a resident uses information from others for his own training or makes his content available to others. A major factor is the scientifically often inferior quality of the content provided on social media platforms, about which more than 50% of respondents in this study also expressed concerns [34]. This begins with the fact that it is sometimes not clear who the author of the information presented is. In addition, there is no scientific control in the sense of peer-review procedures, and even less can it be guaranteed that the patient cases shown have been treated on an evidence-based level. Any user can share any information, even incorrect, without this having to entail any consequences. Frequently, conflicts of interest are not specified, so that economic interests or contributions that are used for marketing or advertising purposes cannot always be identified with certainty [35, 36]. It should, therefore, be recommended that residents first critically examine any information they receive via social media and read the accuracy of this in specialist literature or in medical databases or check for conflicts of interests before adopting the practice in their own portfolio. When posting medical content, another important point is the patient's rights, in particular patient privacy, which must be protected in any case. Only information about a patient to which the patient has consented may be published or shared on social media. When obtaining the patient's consent, it must be explicitly stated that the publication of patient data or photos on a social media platform is planned. Especially in cases in the maxillofacial area, there is always the possibility that the patient can be identified on the published photos [36, 37]. Furthermore, it should be noted that the doctor should strictly distinguish between a private and a professional account, since the content posted has an influence on the reputation and future career of the doctor himself as well as on the reputation of his related medical institution. In addition, care should be taken to ensure a professional doctor-patient relationship [36–38]. To avoid these concerns, there are guidelines and action recommendations for the use of social media from various international and national professional societies, such as the handout of the German Medical Association "Doctors on Social Media," which can support doctors, institutions and patients to work with social media in the correct way [39–41]. It is interesting and worrying that 43% of participants had no concerns at all about using medical content on social media. Additionally, 97% had not received any further education of social media use in a professional context. This shows a clear lack of education in this increasingly important field of social media and should be addressed soon in order to protect the users themselves as well as their patients from moral and legal consequences [37].

## Limitations

With *n* = 64 participants our study was slightly underpowered which could be limitation to the conclusions drawn. Moreover, it remains unclear whether our results are transferable to OMFS residents world-wide since the usage of specific social media platforms strongly differs from country to country.

## Conclusion

Digital and innovative medical education concepts play an important role in OMFS training and education and are highly accepted by OMFS residents. This can help to preserve the high educational standards within this surgical specialty and to acquire well-trained future OMF surgeons. Academic institutions and societies should implement SM to their educational activities as a promising addition to established training and teaching concepts.

## Data Availability

The datasets used and analysed during the current study are available from the corresponding author on reasonable request.

## Abbreviations

CME: Continuing medical education
OMFS: Oral- and Maxillofacial Surgery
SM: Social Media

## Footnotes

1 The Young Forum represents the interests of OMFS residents throughout Germany and provides an integrative network for the exchange of information on clinical, scientific, didactic and social aspects.
